# Mid-trimester prediction of spontaneous preterm birth with automated cervical quantitative ultrasound texture analysis and cervical length: a prospective study

**DOI:** 10.1038/s41598-021-86906-8

**Published:** 2021-04-02

**Authors:** Xavier P. Burgos-Artizzu, Nuria Baños, David Coronado-Gutiérrez, Julia Ponce, Brenda Valenzuela-Alcaraz, Ana L. Moreno-Espinosa, Laia Grau, Álvaro Perez-Moreno, Eduard Gratacós, Montse Palacio

**Affiliations:** 1 Transmural Biotech S. L, Barcelona, Spain; 2 BCNatal - Barcelona Center for Maternal-Fetal and Neonatal Medicine, Hospital Clínic and Hospital Sant Joan de Deu, University of Barcelona, Barcelona, Spain; 3 Institut D’Investigacions Biomèdiques August Pi I Sunyer, IDIBAPS, Barcelona, Spain; 4 Center for Biomedical Research on Rare Diseases (CIBER-ER), Barcelona, Spain

**Keywords:** Medical imaging, Predictive markers, Computer science

## Abstract

The objective of this study was to evaluate a novel automated test based on ultrasound cervical texture analysis to predict spontaneous Preterm Birth (sPTB) alone and in combination with Cervical Length (CL). General population singleton pregnancies between 18 + 0 and 24 + 6 weeks’ gestation were assessed prospectively at two centers. Cervical ultrasound images were evaluated and the occurrence of sPTB before weeks 37 + 0 and 34 + 0 were recorded. CL was measured on-site. The automated texture analysis test was applied offline to all images. Their performance to predict the occurrence of sPTB before 37 + 0 and 34 + 0 weeks was evaluated separately and in combination on 633 recruited patients. AUC for sPTB prediction before weeks 37 and 34 respectively were as follows: 55.5% and 65.3% for CL, 63.4% and 66.3% for texture analysis, 67.5% and 76.7% when combined. The new test improved detection rates of CL at similar low FPR. Combining the two increased detection rate compared to CL alone from 13.0 to 30.4% for sPTB < 37 and from 14.3 to 42.9% sPTB < 34. Texture analysis of cervical ultrasound improved sPTB detection rate compared to cervical length for similar FPR, and the two combined together increased significantly prediction performance. This results should be confirmed in larger cohorts.

## Introduction

Ultrasound measurement of cervical length (CL) is the most accurate predictor of sPTB and of common use worldwide to predict pregnant women at risk. The shorter the cervix, the higher the risk^1^. Women with short CL can be treated with progesterone to reduce the risk of sPTB^2,3^. However, its value for population-wide screening in low risk pregnant women remains controversial mainly because of the low sensitivity and low prevalence of short CL^4–7^. A recent prospective observational cohort study including 9410 nulliparous women with singleton pregnancies, concluded that the low predictive accuracy for sPTB < 37 and < 32 weeks of sonographic CL < 25 mm between 16^+0^ and 22^+6^ (AUC 0.53 and 0.61, respectively) do not support routine use of these test in such women^8^. Among the potential reasons for limited performance of sonographic CL could be a limited inter-observer and intra-observer reproducibility, particularly when cut-offs to classify women as high or low risk are used^9^. The development of methods that identify cervical changes preceding sPTB with less variability than CL could improve prediction rates and maximize the impact of preventive measures such as progesterone treatment.

Despite the multifactorial nature of sPTB, cervical remodeling may precede the clinical onset of the syndrome in a proportion of cases^10^. In turn, it has been hypothesized that inflammation, another cause of sPTB^11^ can also cause CL remodeling^12^. Quantitative texture analysis is a powerful technique to extract information from medical images and quantify tissue changes. Taking into consideration the scientific evidence supporting microstructural changes in the composition of the uterine cervix throughout a normal pregnancy^10,13,14^ and in sPTB, in a previous preliminary study^15^ we evaluated the predictive value of a previous quantitative ultrasound analysis system based on a manually selected cervix area. Results showed predictive accuracies similar to those obtained with CL.

The objective of this study was to evaluate for the first time the performance of a novel, improved test based on fully automated quantitative ultrasound analysis, when used routinely at 20–22 weeks ultrasound to predict sPTB < 34 + 0 and < 37 + 0 weeks. The predictive performance was compared with that of ultrasound cervical length, measured as part of routine clinical practice during the ultrasound study. The results when combining both tools was also evaluated.

## Methods

### Patient recruitment and image acquisition protocol

This was a prospective study on singleton pregnancies assessed between 18 + 0 and 24 + 6 weeks’ gestation at BCNatal (Hospital Clinic and Hospital Sant Joan de Deu, Barcelona) from July 2018 to February 2019. At each hospital, one ultrasound room was selected and all eligible pregnant women attending for routine mid trimester ultrasound were proposed participation. Women with sPTB risk factors (history of sPTB or miscarriage ≥ 16 weeks, and cervical intervention or Müllerian malformation) were included unless they had already received treatment (progesterone, cervical cerclage or cervical pessary) to prevent sPTB. The main clinical outcomes of the study were the occurrence of sPTB before 34 + 0 or 37 + 0 weeks. Preterm births for fetal or maternal indication, including induction of labor (IOL) for preterm prelabor rupture of membranes (PPROM) were excluded**.** Information on baseline demographic characteristics and obstetric history were collected. Perinatal outcomes were retrieved from hospital files. Gestational age was calculated based on crown–rump length measured on first-trimester ultrasound. The study protocol was approved by the local Ethics Committee of the Hospital Clínic (ID HCB 2014/0089) and Hospital Sant Joan de Déu (ID PIC-147-15), all methods were performed in accordance with the relevant guidelines and regulations and all pregnant women provided written informed consent.

Similarly to our previous preliminary study^15^, one image of the uterine cervix was obtained for each woman. Images were acquired by experienced sonographers performing routine screening ultrasound. A sagittal view of the cervix was obtained. The internal and external os, as well as the cervical canal, were identified and the entire cervical structure visualized, avoiding zooming and using only the depth function. Shadows and saturations were avoided as possible. Any post-processing functions, such as speckle reduction imaging or smoothing, were disabled. Cervical length was measured online by the sonographer performing the examination and saved together with baseline demographic and perinatal outcomes. Gain was at the discretion of the physician. Images were acquired with Siemens Sonoline Antares (Siemens Medical Systems, Malvern, PA, USA) or VolusonE6 (GE Medical Systems, Zipf, Austria) ultrasound machines, with a 2–10-MHz vaginal probe by different operators. All study images were stored in the original Digital Imaging and Communication in Medicine (DICOM) format for further analysis.

It is important to note that following our protocol, progesterone was administered only if cervical length was < 20 mm or < 25 mm in high-risk patients (those having had a previous preterm birth).

### Cervical ultrasound image automated processing

**Figure 1:**
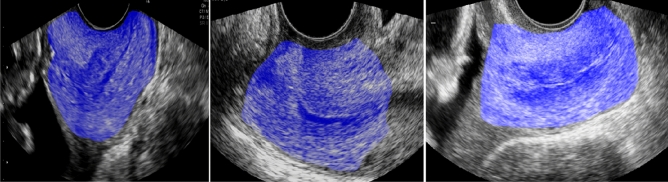
Example images and the automatic ROI delineation performed by QUANTUSPREMATURITY.

DICOM images were processed using the novel automated quantitative test, called QUANTUSPREMATURITY and available online (www.quantusprematurity.org). The tool is very simple to use as it only requires the image to be uploaded online. Then, the test automatically delineates a region of interest (ROI) containing the entire cervix, see Fig. 1 and calculates a sPTB probability risk score, which is returned to the user in the form of a Portable Document Format (PDF) clinical report. This report contains the prematurity risk score estimated by the test, which was stored for further analysis.

### Statistical analysis

Statistical analyses were performed using MATLAB (Mathworks, USA). Statistical differences between demographic and clinical outcomes were calculated using the standard mean difference between cases and control values. To measure statistical significance of these differences, we used null hypothesis significance testing. We used fisher exact test for discrete variables and t-test for continuous, normally distributed values.

The performance of QUANTUSPREMATURITY was then compared with cervical length to predict sPTB at < 34 and < 37 weeks. First, the test risk score and CL value in mm were used to draw Receiver Operator Characteristic (ROC) curves and compute full Area Under the Curve (AUC) with their 95% Confidence Intervals. Then, the ROC curves were used to establish the optimal cutoff points as those maximizing accuracy. Detection rate, false positive rate (FPR), positive and negative predictive values (PPV and NPV) and positive and negative likelihood ratios (LR+ and LR−) were calculated with their 95% Confidence Intervals using these cut-off points. The same process was repeated for the combination of both tools, combining QUANTUSPREMATURITY after binarization using the aforementioned cut-off points with CL at three cutoff points (25, 28 and 30 mm). The combined algorithm was a simple OR gate, predicting negative sPTB when both tests were negative and positive otherwise (only one or both were positive).

We then tested the independence between the test risk score and CL values in mm, by computing the pearson correlation coefficient between them and its associated statistical significance level of correlation. Finally, to compute statistical significance of the differences in prediction performance between the two tools, we used the combined Wald test which tests if there are statistical differences in both sensitivity and specificity.

### Ethical approval

The study protocol used was approved by the local Ethics Committee of the Hospital Clínic (ID HCB 2014/0089) in March 2014 and Hospital Sant Joan de Déu (ID PIC-147-15) in March 2015, and all pregnant women provided written informed consent.

## Results

A total of 633 consecutive patients were included in the study. After reviewing the perinatal outcomes, 7 cases (1.1%) of sPTB < 34 + 0 weeks and 23 cases (3.6%) of sPTB < 37 + 0 were identified. Demographic characteristics, cervical measurements and perinatal outcomes for the women included in the study are shown in Table 1. Maternal baseline characteristics did not differ between term and sPTB pregnancies. Only CL, GA at delivery, spontaneous onset of labor, birthweight and progesterone showed statistical significant differences between women who delivered preterm vs those who delivered at term. Average CL was slightly smaller for preterm (38.9 mm) than at term (40.8 mm) pregnancies. Prevalence of CL <  = 25 mm was 0.8% and was higher among women who gave birth preterm compared to term pregnancies (8.7% vs 0.5%). Please note that only one patient received progesterone and still delivered preterm, therefore progesterone did not impact outcome.

**Table 1:** General characteristics of the population.

	Total N = 633	Term birth N = 610 (96.4%)	sPTB < 37 N = 23 (3.6%)	sPTB < 34 N = 7 (1.1%)	*p* value *	*p* value **
Maternal age (years)	33.5 (5.3)	33.5 (5.2)	33.5 (6.0)	34.5 (5.7)	0.98	0.61
BMI	24.0 (4.1)	24.0 (4.0)	25.2 (5.5)	23.1 (3.1)	0.16	0.54
Caucasian	434 (68.6%)	419 (68.7%)	15 (65.2%)	5 (71.4%)	0.73	0.87
Tobacco use	49 (7.7%)	45 (7.4%)	4 (17.4%)	2 (28.6%)	0.08	0.04
Nuliparous	332 (52.4%)	322 (52.8%)	10 (43.5%)	4 (57.1%)	0.38	0.80
PPTB37	13 (2.1%)	13 (2.1%)	0 (0.0%)	0 (0.0%)	0.48	0.70
PPTB34	9 (1.4%)	9 (1.5%)	0 (0.0%)	0 (0.0%)	0.56	0.75
GA (weeks)	20.8 (1.0)	20.8 (1.0)	20.7 (0.9)	20.9 (0.6)	0.67	0.73
CL (mm)	40.7 (7.5)	40.8 (7.5)	38.9 (8.9)	35.7 (8.2)	0.25	0.08
CL < = 25 mm	5 (0.8%)	3 (0.5%)	2 (8.7%)	1 (14.3%)	0.00	0.00
Uterine factor	30 (4.7%)	26 (4.3%)	4 (17.4%)	0 (0.0%)	0.27	0.55
Spontaneous onset of labour	360 (56.9%)	337 (55.2%)	23 (100%)	7 (100%)	0.00	0.02
Vaginal delivery	472 (74.6%)	457 (74.9%)	15 (65.2%)	5 (71.4%)	0.30	0.85
GA at delivery (weeks)	39.1 (3.1)	39.4 (2.7)	32.9 (5.1)	26.9 (6.0)	0.00	0.00
Birthweight (g)	3276.0 (475.4)	3300.0 (450.6)	2641.9 (660.2)	2509.0 (1052.6)	0.00	0.00
Progesterone	1 (0.1%)	0	1 (4%)	1 (14%)	0.00	0.00

Data given as: mean (std) or n (%). *BMI* body mass index, *GA* gestational age, *sPTB* spontaneous preterm birth, *CL* cervical length, *PPTB37* previous preterm birth at week 37, *PPTB34* previous preterm birth at week 34. *Uterine Factor* Uterine conisation or malformation. *p* value, fisher exact test (discrete variables) or t-test (continuous variables) significance value between cases and controls. Progesterone, Patient received progesterone.

*Comparison between term group and sPTB < 37 weeks.

**Comparison between term group and sPTB < 34 weeks.

**Figure 2:**
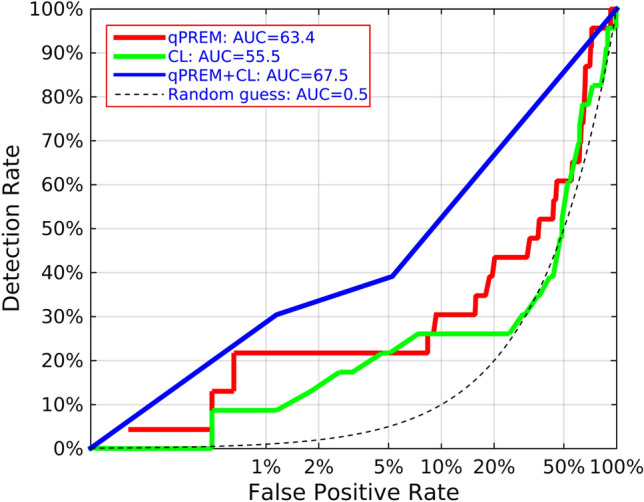
ROC curve for sPTB < 37 weeks. qPREM (Red) = automated cervical texture analysis tool. CL(Cyan): Cervical Length. qPREM + CL (blue) = both tools combined. Black dashed curve = random guess. X axis is shown in logarithmic scale to focus on low False Positive Rates, the only useful from a clinical perspective for sPTB prediction during mid-trimester screening of general population.

**Figure 3:**
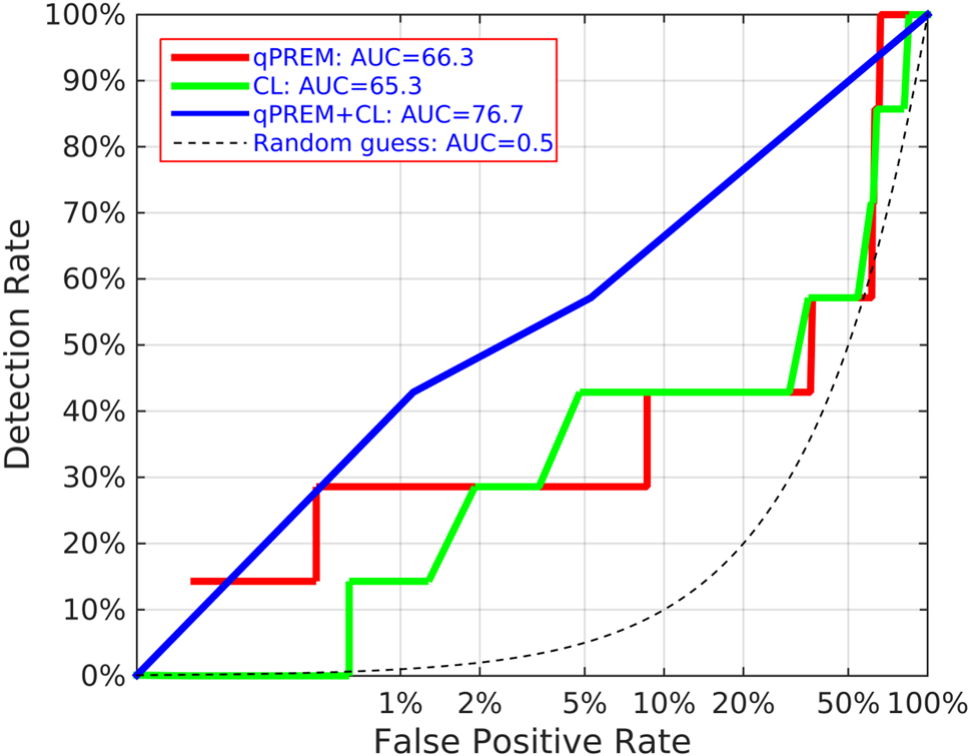
ROC curve for sPTB < 34 weeks. qPREM (Red) = automated cervical texture analysis tool. CL (Cyan): Cervical Length. qPREM + CL (blue) = both tools combined. Black dashed curve = random guess. X axis is shown in logarithmic scale to focus on low False Positive Rates, the only useful from a clinical perspective for sPTB prediction during mid-trimester screening of general population.

Figures 2 and 3 show the ROC curves for both QUANTUSPREMATURITY, CL and the two combined for predicting sPTB < 37 + 0 and < 34 + 0 weeks, respectively. The ROC curves are plotted using a logarithmic X axis to focus on the low false positive rates, the only valid from a clinical perspective given the low prevalence of sPTB. AUC for sPTB prediction before weeks 37 and 34 respectively were as follows: 55.5%(± 12.6%) and 65.3%(± 24.6%) for CL, 63.4%(± 8.0%) and 66.3%(± 20.7%) for texture analysis, 67.5%(+ -10.9%) and 76.7%(+ -20.0%) when combined. The quantitative ultrasound test improved the detection rate of CL for false positive rates values below 5% for both sPTB < 37 + 0 and < 34 + 0 weeks. When combined together, detection rates are improved significantly across any false positive rate.

Tables 2 and 3 show the results for sPTB < 37 + 0 and < 34 + 0 prediction in terms of Detection rate, False positive rate, PPV, NPV, LR+ and LR− using the optimal cut-off points in terms of accuracy (CL optimal cut-off point was 25 mm, in accord with most studies). The test predicted sPTB < 37 + 0 and < 34 + 0 weeks with a 21.7% (± 2.7%) and 28.6% (± 6.6%) detection rate at false positive rates of 0.6% (± 0.1%) and 0.5% (± 0.1%) respectively. In comparison, CL predicted sPTB < 37 + 0 and < 34 + 0 weeks with a 8.7% (± 2.0%) and 14.3% (± 5.1%) detection rate with the same false positive rates. Combining both tests together resulted in detection rates of 30.4% (± 3.1%) and 42.9% (± 7.3%) for false positive rates of 1.1% (± 0.2%) and 1.1% (± 0.1%) respectively (using again CL with a 25 mm cutoff).

**Table 2:** Results for sPTB < 37.

Method	AUC	Accuracy	Detection rate	FP rate	PPV	NPV	LR +	LR −
qPREM	63.4 (± 8.0)	611/633 (96.5% ± 0.2%)	5/23 (21.7% ± 2.7%)	4/633 (0.6% ± 0.1%)	5/9 (55.6% ± 4.6%)	606/624 (97.1% ± 0.1%)	33.2 (± 7.3)	0.8 (± 0.0)
CL < = 25 mm	55.5 (± 12.6)	609/633 (96.2% ± 0.1%)	2/23 (8.7% ± 2.0%)	3/633 (0.5% ± 0.1%)	2/5 (40.0% ± 4.6%)	607/628 (96.7% ± 0.1%)	17.7 (± 3.6)	0.9 (± 0.0)
qPREM + CL < = 25 mm	67.5 (± 10.9)	610/633 (96.4% ± 0.2%)	7/23 (30.4% ± 3.1%)	7/633 (1.1% ± 0.2%)	7/14 (50.0% ± 4.4%)	603/619 (97.4% ± 0.1%)	26.5 (± 5.1)	0.7 (± 0.0)

N = 633, 23 cases, 610 controls (prevalence 3.6%).

*qPREM* QUANTUSPREMATURITY, *CL* cervical length, *FP Rate* false positive rate, *PPV* positive predictive value, *NPV* negative predictive value, *LR* + positive likelihood ratio, *LR − *negative likelihood ratio.

**Table 3:** Results for sPTB < 34.

Method	AUC	Accuracy	Detection rate	FP rate	PPV	NPV	LR +	LR −
qPREM	66.3 (± 20.7)	625/633 (98.7% ± 0.1%)	2/7 (28.6% ± 6.6%)	3/633 (0.5% ± 0.1%)	2/5 (40.0% ± 11.8%)	623/628 (99.2% ± 0.1%)	59.6 (± 36.6)	0.7 (± 0.1)
CL < = 25 mm	65.3 (± 24.6)	623/633 (98.4% ± 0.2%)	1/7 (14.3% ± 5.1%)	4/633 (0.6% ± 0.1%)	1/5 (20.0% ± 8.3%)	622/628 (99.0% ± 0.1%)	22.4 (± 11.8)	0.9 (± 0.1)
qPREM + CL < = 25 mm	76.7 (± 20.0)	622/633 (98.3% ± 0.2%)	3/7 (42.9% ± 7.3%)	7/633 (1.1% ± 0.1%)	3/10 (30.0% ± 4.9%)	619/623 (99.4% ± 0.1%)	38.3 (± 7.8)	0.6 (± 0.1)

N = 633, 7 cases, 626 controls (prevalence 1.1%).

*qPREM* QUANTUSPREMATURITY, *CL* cervical length, *FP Rate* false positive rate, *PPV* positive predictive value, *NPV* negative predictive value, *LR* + positive likelihood ratio, *LR − *negative likelihood ratio.

Pearson correlation coefficient between the test risk score computed by QUANTUSPREMATURITY and CL values in mm was very low (pearson correlation = 1%, associated *p*-value = 0.78) indicating a clear statistical difference between the values output of both tools.

When used to compare qPREM alone versus CL for prediction of sPTB < 37 + 0 and < 34 + 0 weeks, Wald test resulted in higher than 0.05 *p*-values (*p* = 0.17 and *p* = 0.47 respectively). However, when used to compare the combination of both tools vs CL alone, *p*-values were smaller than 0.05 (*p* < 0.01 and *p* = 0.04 respectively). This indicates, as evident also from Tables 2 and 3, that differences in performance between the tool on its own and CL are small but that the improvement is clearly significant when both are combined together.

## Discussion

### Main findings

This study evaluates for the first time and prospectively in a general population, the performance of a novel test based on quantitative analysis of cervical texture to predict sPTB, called QUANTUSPREMATURITY The study provides evidence that quantitative analysis of ultrasound cervical texture at 20–22 weeks improves sPTB prediction before 34 + 0 and 37 + 0 weeks in comparison with standard ultrasound CL measurement. The test improves detection rates at low false positive rates values and is fully automated. Moreover, the test is independent and complementary to CL and both can be combined together for a significant increase to detection rates at similar false positive rates.

In the general population evaluated in this study, the new test improved the sensitivity of CL for any false positive rate (see Fig. 2). It was able to increase detection rate from 8 to 21% for prediction of sPTB < 37 weeks and from 14 to 28% for prediction of sPTB < 34 weeks maintaining false positive rates below 1% (Tables 2 and 3). Moreover, when combined together with CL, detection rates were further improved to 30% for prediction of sPTB < 37 weeks and to 42% for prediction of sPTB < 34 weeks, assuming a false positive rate of only 1.1%.

This is a completely different study from the previous preliminary study presented by our group^15^. Firstly, this study was conducted including pregnant women collected prospectively during routine mid-trimester screening, compared to the case–control nature of the previous study. These women were not used for the previous study (this is a completely new dataset) and this time no women from the preterm birth unit were included. Moreover, the test evaluated in this study was completely different. The new QUANTUSPREMATURITY test is fully automated as it automatically identifies and segments the cervix from the cervical ultrasound image, further reducing the need for manual intervention in the evaluation (see Fig. 1). This ensures full repeatability of the result given the same image, thus overcoming the limitations of CL manual measurement, which has been shown to be highly operator-dependent, particularly when cut-off points are used^9^. Finally, in this study we report the results of a final “closed” algorithm, available online, compared to the previous study where several potential prototypes were used due to the use of kfold cross-validation. Finally, the combination of the automated test together with CL is evaluated for the first time.

### Interpretation (in light of other evidence)

From a clinical perspective, these results suggest that automated quantitative cervical assessment could improve the performance of currently used CL measurement. The reasons for improved prediction might lie in the ability of texture analysis to pick up extremely subtle changes associated with early cervical remodeling and eventually increasing sPTB risk that escape the human eye or CL measurement. It is also plausible that the factors involved in inflammation, whose relationship with preterm delivery has been established^12^, do induce changes in the cervical level to some extent. Additionally, automated evaluation by definition reduces the variability of subjective measurements. If the results of this study are confirmed, they would justify incorporation of automated measures for the screening of sPTB. A screening system increasing the current detection rates reported for CL measurement could impact in current policies and recommendations.

Although the new tool improves detection rate compared to the use of the standard use of cervical length, the detection rate is still low. This might be explained because preterm birth is a multifactorial syndrome in nature^11^ and therefore achieving a very high detection rate and PPV with a single test might be an unrealistic goal. However, the detection rate improvement of the new tool with respect to CL might help to be able to better select a group of women in which strategies as progesterone, pessary or cerclage might be effective, without the burden of a high positive rate.

### Strengths and limitations

This study has several strengths. It was performed prospectively on routine mid-trimester screening patients. The sPTB prevalence is in line with that reported for the general population of pregnant women in Spain^16^ (1.1% < 34 weeks and 3.6% < 37 weeks). Images used were taken during routine practice by several operators using different ultrasound machines, therefore under the conditions of a real clinical setting. Finally, the average CL measurements and the prevalence of CL < 25 mm was similar to that reported in recent studies on general pregnant women of other European countries^6,7^. Other relevant demographic characteristics such as previous sPTB or prevalence of short CL are also concordant with the latest published data^6,7^.

We acknowledge a number of limitations. First, the number of preterm births is relatively limited. This is partially due to the strict definition of spontaneous preterm birth in our protocol. A few more cases could have been added but we decided to exclude them to avoid overestimating the predictive capacity of the tool. More specifically, four cases of PPROM who underwent an IOL and delivered between weeks 34–37 were excluded since reviewing each case individually we considered them to be similar to preterm delivery after IOL indicated for other medical interventions (as for IUGR or preeclampsia).

On the other hand, although number of preterm births < 34 weeks was very low, the outcome of sPTB < 37w is still relevant. Considering that 85.9% % of the moderate and late preterm deliveries occur between 34 and 37 weeks, detection and potential treatment of these cases may well impact on perinatal results and economic health burden^17^. However, performance of the test after preventive strategies as progesterone or pessary remains to be assessed.

Finally, we acknowledge that these results should be validated externally through a large multicenter prospective cohort study.

## Conclusion

Automated texture analysis of the cervix alone or in combination with CL predicted sPTB at < 34 and < 37 weeks with higher detection rates compared with conventional measurement of CL. If confirmed, these results would support the addition of automated texture analysis to improve prediction of sPTB when mid-trimester universal cervical screening is used.
